# Dual regression physiological modeling of resting-state EPI power spectra: Effects of healthy aging

**DOI:** 10.1016/j.neuroimage.2018.01.011

**Published:** 2019-02-15

**Authors:** Olivia Viessmann, Harald E. Möller, Peter Jezzard

**Affiliations:** a Wellcome Centre for Integrative Neuroimaging, FMRIB Division, Nuffield Department of Clinical Neurosciences, University of Oxford, Oxford, UK; b Max Planck Institute for Human Cognitive and Brain Sciences, Stephanstraße 1a, 04103 Leipzig, Germany

**Keywords:** EPI, Dual regression, fMRI, Physiological noise, Cardiac pulsatility, Healthy aging

## Abstract

Aging and disease-related changes in the arteriovasculature have been linked to elevated levels of cardiac cycle-induced pulsatility in the cerebral microcirculation. Functional magnetic resonance imaging (fMRI), acquired fast enough to unalias the cardiac frequency contributions, can be used to study these physiological signals in the brain. Here, we propose an iterative dual regression analysis in the frequency domain to model single voxel power spectra of echo planar imaging (EPI) data using external recordings of the cardiac and respiratory cycles as input. We further show that a data-driven variant, without external physiological traces, produces comparable results. We use this framework to map and quantify cardiac and respiratory contributions in healthy aging. We found a significant increase in the spatial extent of cardiac modulated white matter voxels with age, whereas the overall strength of cardiac-related EPI power did not show an age effect.

## Highlights

• We present a dual regression method to model power spectra of ultra-fast EPI data to map and quantify physiological contributions.
• The method was used to study cardiac and respiratory contributions to the EPI data in healthy aging.
• We found a significant increase in the spatial extent of cardiac modulated voxels in white matter with age.
• The strength of cardiac-related EPI power did not show an age effect.

## Introduction

### Physiological noise in broadband rs-fMRI

Blood-oxygenation level dependent (BOLD) functional magnetic resonance imaging (fMRI), in particular echo planar imaging (EPI), has traditionally been used to study neuronal activity in the resting state (rs-fMRI) or whilst performing a task. The fMRI time series is a composite of neurovascular and additional noise contributions, the latter of which are often regarded as unwanted and are removed from the data in post processing (see (Bulte and Wartolowska, 2017; Murphy et al., 2013), and (Birn, 2012) for a review). Noise is categorised into thermal and physiological noise, where the former can be modelled as white noise but the latter is a non-random signal. The most prominent physiological noise sources are respiration- and cardiac cycle-induced signal fluctuations. Cardiac cycle-related signals are more pronounced around major blood vessels (Dagli et al., 1999), sulci and perivascular cerebrospinal fluid (CSF) spaces, but are also measurable in grey and white matter (Shmueli et al., 2007, Chang and Glover, 2009). Respiratory-related fluctuations are dominantly spread across the peripheral areas (Tong and Frederick, 2014).

With the technical developments of in-plane (partial Fourier (Feinberg et al., 1986), GRAPPA (Griswold et al., 2002) and SENSE (Pruessmann et al., 1999)) and through-plane (simultaneous multi-slice (SMS)/multiband (Moeller et al., 2010, Feinberg et al., 2010, Feinberg and Setsompop, 2013)) acceleration, repetition times (TR) can be reduced to a few hundreds of milliseconds whilst still achieving reasonable coverage and spatial resolution. As such, EPI time courses can be resolved beyond the cardiac frequency regime. The question of whether vascular properties are reflected in the cardiac frequency band turns what is usually regarded as a nuisance artifact into a signal in its own right.

### Cerebral cardiac pulsatility

Age-related wall stiffening of the major arteries impedes the transformation of pulsatile arterial blood flow to a steady flow in the capillary bed (see (O'Rourke and Hashimoto, 2007) for a review). The reduced ability of the arteriovasculature to dilate and constrict is thought to increase flow pulsatility^1^ in the cerebral microcirculation. Pulsatility measures have further been linked to MRI-derived measures. Increased middle-cerebral artery (MCA) flow pulsatility has been found in patients with white matter hyperintensities (Webb et al., 2012). To specifically measure effects in tissue, Makedonov et al. (2013). introduced a “cardiac pulsatility metric” and a “physiological noise metric” based on EPI power spectra. Both metrics showed significant differences in white matter with age, small vessel disease and the latter metric also in Alzheimer's disease (Makedonov et al., 2016).

### Methods to model cardiac signal contributions

Methods to identify physiological signal components can be classified broadly into those that require external physiological recordings and those that are data-driven. If external recordings are available it is most common to perform RETROICOR (Glover et al., 2000). This method synchronizes the acquisition timing of each fMRI volume to the phase of the subject's cardiac and respiratory cycle to fit a Fourier series to each voxel's time series. The Fourier coefficients subsequently serve as regressor maps. This approach is insensitive to signal fluctuations where there is a variation in cardio-respiratory frequencies, and requires an accurate synchronisation of scanner triggers and physiological recording (extra care is needed in the case of SMS/multiband). Data-driven approaches are dominated by independent component analysis (ICA), where the 4D data set is decomposed into a set of components, each consisting of a 3D spatial map of weights and an associated time course (McKeown et al., 1998, Beckmann et al., 2005). These can subsequently be sorted into signal with neuronally- or physiologically-related origin by visual inspection or by using trained classifiers (Salimi-Khorshidi et al., 2014, Griffanti et al., 2014). All of the above methods produce regressor maps that could be used to quantify cardiac components. However, it is not straightforward to decide on a set of components and how to normalise within and across subjects.

### Aim of this work

The availability of analysis frameworks to map and quantify cardiac-related EPI signals is limited to date. To disentangle respiratory and cardiac-driven physiological from neurovascular fluctuations, we acquired high-temporal resolution rs-fMRI data with a TR of 328 ms, corresponding to a Nyquist frequency for the power spectrum of 1.52 Hz.

**Fig. 1:**
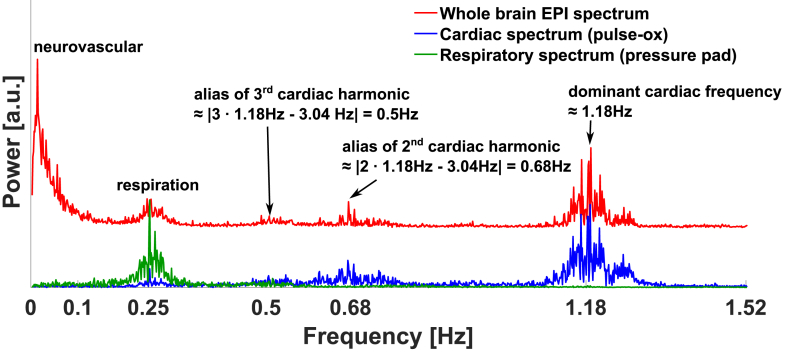
Exemplar whole brain rs-EPI power spectrum (red) and the power spectra of the cardiac (blue) and respiratory trace (green) from external measurements (pulse-ox at the finger and a pressure pad on the chest). The external physiological signals are sub-sampled to the EPI TR. Above the low-frequency neurovascular range (≲0.1 Hz) the EPI spectrum closely matches the physiological spectra. The cardiac spectrum shows aliases of the second and third harmonics of the dominant cardiac frequencies (according to $f_{\text{alias}}=|f_{\text{true}}-n\cdot f_{\text{sample}}|,n\in \mathbb{N}$).

Fig. 1 shows a representative average whole brain^2^ BOLD spectrum from such a scan during the resting state. The externally measured cardiac and respiratory power spectra (sub-sampled to TR) are displayed in the same graph. In the low-frequency regime (≲0.1 Hz) the spectrum is dominated by neurovascular BOLD fluctuations and other low-frequency sources, most likely blood flow and volume changes due to end-tidal changes in partial pressure of CO_2_ (PET-CO_2_) (Wise et al., 2004), bulk motion and potentially vasomotor fluctuations (Kiviniemi et al., 2000). Above this band, the EPI power spectrum appears highly correlated with the external physiological spectra. Hence, we make the following assumption: The BOLD power spectrum above 0.2 Hz in each voxel can be modelled as a linear combination of a constant thermal noise baseline, plus a cardiac and respiratory power spectrum in a general linear model (GLM). We propose an iterative dual regression scheme to identify voxels that exhibit significant cardiac and respiratory-related power. The spatial extent and strength of these fluctuations can then be assessed. We used externally recorded cardiac and respiratory traces as initial explanatory variables. As this method is based on the power spectrum it does not require slice-timing correction or scanner trigger synchronisation. Respiratory traces are usually recorded with a respiratory belt or pad that measure pressure changes from chest movement during inhalation and exhalation. These amplitude traces are, however, a sub-optimal representation of the multiple factors that affect the EPI times series, from susceptibility variations with respiratory volume and rate changes (Birn et al., 2008, Chang and Glover, 2009) as well as related PET-CO_2_ changes. Similarly, the measurement of pulse-oximetry (pulse-ox) data do not exactly resemble the cardiac cycle-induced power spectra in the brain. Even within the brain, in particular between different tissues, we expect differences in the physiologically-related EPI power contributions. To approach the “true” cardio-respiratory spectra in each tissue we ran an iterative dual regression, where the externally measured physiological spectra serve as the initial “guess”. Additionally, we explore a data-driven dual regression approach that solely recreates the cardio-respiratory spectra from the EPI time series data themselves.

We then use this framework to test if the cardiac contributions to the EPI power spectrum show an age effect in healthy adults. Specifically, we compare the strength and spatial extent of those contributions in normal ageing.

## Methods

### The spectral GLM

**Fig. 2:**
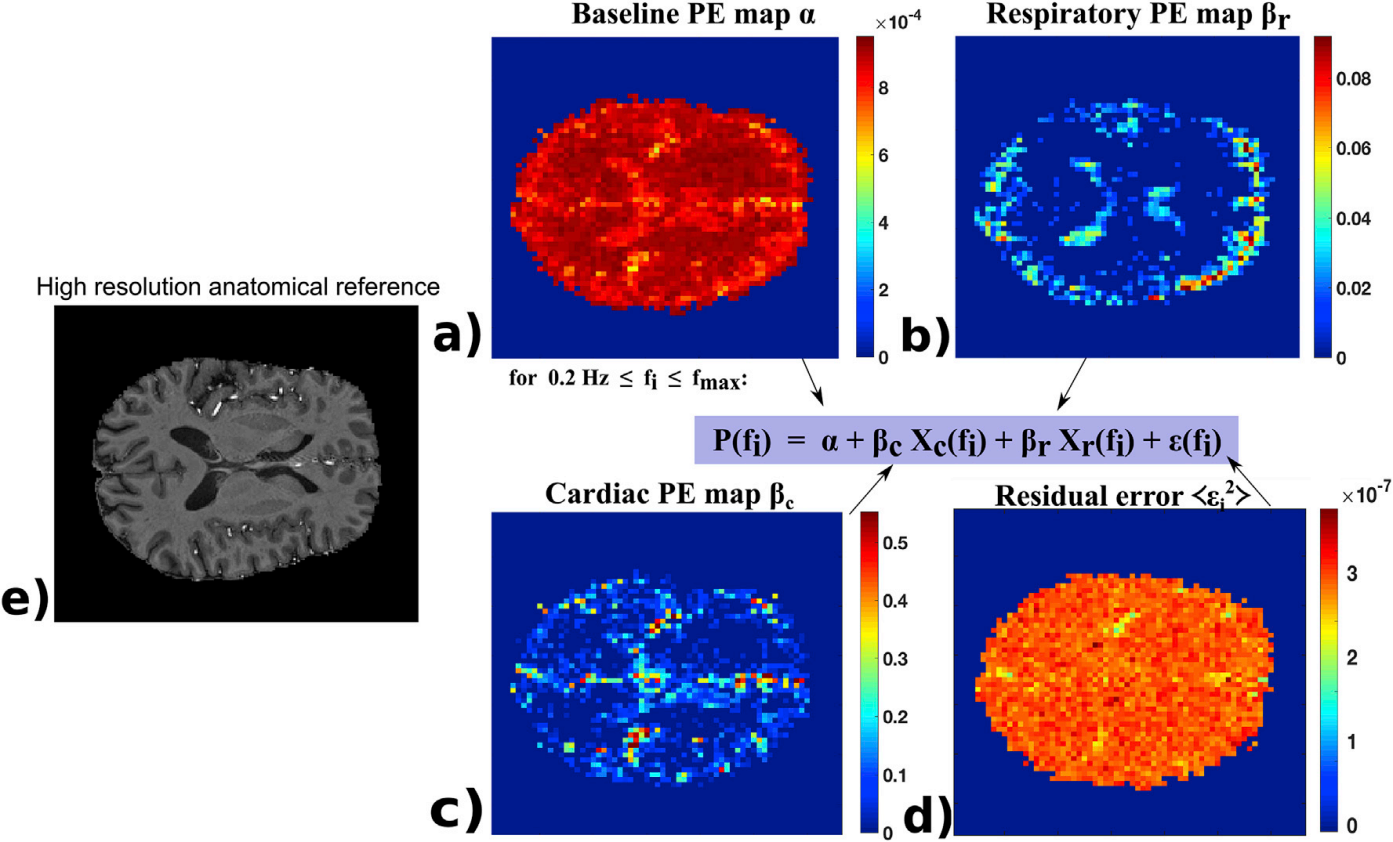
PE maps from an exemplar subject. The noise baseline map *α* in (a) fills the entire brain as every voxel has a constant thermal noise level. The respiratory map (b) is more pronounced in the periphery and the cardiac map (c) is more prevalent around arterial structures and narrow CSF-filled perivascular spaces. (d) displays the average residual error and (e) is a high resolution anatomical reference T1w image (manually chosen to match the PE maps that are in lower resolution EPI space).

Let $S\left(t_{i}\right)$ be the BOLD time series in a voxel. The power spectrum *P* of this signal is calculated as the amplitude of its Fourier-transform (FT)

(1) $$s\left(f_{1}\ldots f_{N}\right)=FT\left[S\left(t_{1}\ldots t_{N}\right)\right]$$

(2) $$P\left(f_{1}\ldots f_{N/2}\right)=\sqrt{Re{\left\{s\left(f_{1}\ldots f_{N/2}\right)\right\}}^{2}+Im{\left\{s\left(f_{1}\ldots f_{N/2}\right)\right\}}^{2}}$$

where *N* is the total number of sampled time points. $P\left(f_{i}\right)$ spans from $f_{0}=0$ Hz to the Nyquist frequency $f_{\text{max}}=1/\left(2\text{TR}\right)$. Above a neuronal regime frequency threshold (here $f_{\text{min}}=0.2$ Hz) this can be represented as a GLM of a thermal noise baseline, plus a respiratory, $X_{r}\left(f\right)$, and a cardiac $X_{c}\left(f\right)$, power spectrum. In explicit matrix form this is

(3) $$\overset{\text{EPI power spectrum}}{\overset{︷}{\left[\begin{array}{c} P_{1;f_{min}}\ldots P_{1,f_{max}}\\ \vdots \\ P_{s;f_{min}}\ldots P_{s;f_{max}} \end{array}\right]}}=\overset{\begin{array}{c} \text{baseline}\\ \text{PE map} \end{array}}{\overset{︷}{\left[\begin{array}{c} \alpha_{1}\ldots \alpha_{s} \end{array}\right]}}\cdot \left[\begin{array}{c} 1\\ \vdots \\ 1 \end{array}\right]+\underset{\begin{array}{c} \text{respiratory}\\ \text{PE map} \end{array}}{\underset{︸}{\left[\begin{array}{c} \beta_{r,1}\ldots \beta_{r,s} \end{array}\right]}}\cdot \overset{\begin{array}{c} \text{external}\\ \text{spectrum} \end{array}}{\overset{︷}{\left[\begin{array}{c} X_{r,f_{min}}\\ \vdots \\ X_{r,f_{max}} \end{array}\right]}}+\underset{\begin{array}{c} \text{cardiac}\\ \text{PE map} \end{array}}{\underset{︸}{\left[\begin{array}{c} \beta_{c,1}\ldots \beta_{c,s} \end{array}\right]}}\cdot \overset{\begin{array}{c} \text{external}\\ \text{spectrum} \end{array}}{\overset{︷}{\left[\begin{array}{c} X_{c,f_{min}}\\ \vdots \\ X_{c,f_{max}} \end{array}\right]}}+\underset{\begin{array}{c} \text{residual}\\ \text{error} \end{array}}{\underset{︸}{\varepsilon_{\left[s,f\right]}}}$$

with s={x,y,z} being the spatial dimensions and $\alpha ,\beta_{r},\beta_{c}$ being the parameter estimates (PEs) for the noise baseline, respiration and cardiac spectral power contribution and ε is the residual error. Fig. 2 shows exemplar PE maps from this GLM. The thermal noise baseline map covers the entire brain, whereas cardiac and respiratory contributions are significant in only a subset of voxels. Respiration is more pronounced in the periphery of the brain and cardiac fluctuations are dominant in larger arterial structures and narrow CSF spaces, as expected.

We normalise each voxel's power spectrum between the lower cut-off frequency of 0.2 Hz and $f_{\text{max}}$ to be independent of any neuronal activity-induced bias in the power spectra. Explanatory variables are normalised too:

(4) $$\sum_{f_{\text{min}}=0.2}^{f_{\text{max}}}P\left(f_{i}\right)=\sum_{f_{\text{min}}=0.2}^{f_{\text{max}}}X_{c}\left(f_{i}\right)=\sum_{f_{\text{min}}=0.2}^{f_{\text{max}}}X_{r}\left(f_{i}\right)=1.$$

As such the PEs are inter-dependent. A voxel with no significant cardio-respiratory fluctuations will have a higher baseline *α* value than a voxel with physiological signals. Similarly, two voxels with identical respiratory-related fluctuations but different levels of cardiac-related signal variations will not only differ in $\beta_{c}$, but also in $\beta_{r}$. This normalisation-induced coupling effect needs to be accounted for in the statistical tests. However, omitting the normalisation is not a viable option as it renders a comparison between voxels difficult, and between subjects impossible, due to the variation in absolute signal levels.

### Dual regression

**Fig. 3:**
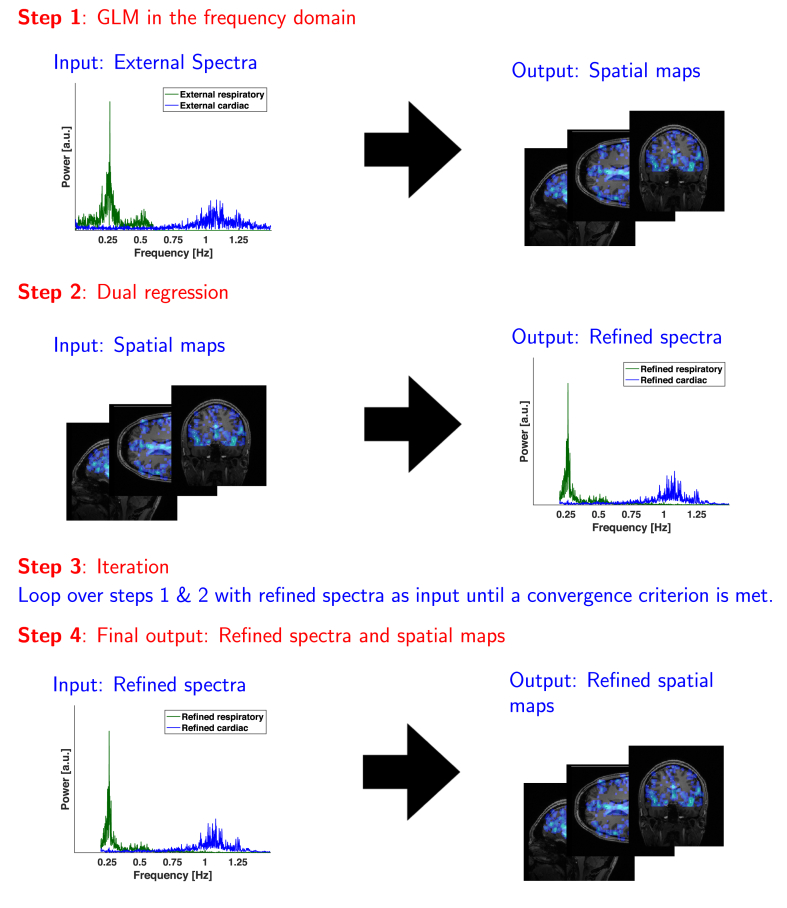
Iterative dual regression scheme to find brain-specific refined spectra and spatial maps of cardio-respiratory fluctuations.

The principle of dual regression has been introduced by Beckmann et al. and Filippini et al (Beckmann et al., 2009, Filippini et al., 2009) in the connectivity analysis of rs-fMRI time series using ICA. They used the group-level derived maps of an ICA component as a set of spatial regressors in a subject-specific GLM to solve for the subject-specific time series associated with each particular map. The resulting time courses are then used as explanatory variables in a re-run of the GLM to further refine the subject-specific maps associated with the particular component. Here, an equivalent strategy can be applied to the spectral data. Fig. 3 gives a schematic overview of the following steps involved:

1. Run a GLM according to the model in Eq. (3) with the externally measured physiological spectra as the initial guess of the explanatory variables.
2. Separate the resulting $\alpha ,\;\beta_{c}$ and $\beta_{r}$ maps along the spatial dimension. Run a second GLM along the frequency dimension (as opposed to the spatial dimension in step 1) to find the “brain-specific”, refined physiological spectra $X_{c}$ and $X_{r}$ and noise baseline constant **1**. Written out explicitly this is
  $$\left[\begin{array}{ccc} P_{1;f_{min}} & \ldots & P_{1,f_{max}}\\ \vdots & & \\ P_{s;f_{min}} & \ldots & P_{s;f_{max}} \end{array}\right]=\left[\begin{array}{ccc} \alpha_{1}^{n} & \beta_{r_{1}}^{n} & \beta_{c_{1}}^{n}\\ \vdots & \vdots & \vdots \\ \alpha_{s}^{n} & \beta_{r_{s}}^{n} & \beta_{c_{s}}^{n} \end{array}\right]\left[\begin{array}{c} 1_{f_{min}}^{n+1}\ldots 1_{f_{max}}^{n+1}\\ X_{r,f_{min}}^{n+1}\ldots X_{r,f_{max}}^{n+1}\\ X_{c,f_{min}}^{n+1}\ldots X_{c,f_{max}}^{n+1} \end{array}\right]+\varepsilon_{\left[s,f\right]}$$
3. Iterate over steps 1 and 2 using the refined spectra from step 2 until a convergence criterion is met.
4. The final outputs are the refined spectra and associated refined spatial maps
  $$P_{\left[s,f\right]}=\alpha_{\left[s,1\right]}^{n}1_{\left[1,f\right]}^{n}+\beta_{c\left[s,1\right]}^{n}X_{c\left[1,f\right]}^{n}+\beta_{r\left[s,1\right]}^{n}X_{r\left[1,f\right]}^{n}+\varepsilon_{\left[s,f\right]}$$

#### Data-driven dual regression

Data-driven reconstruction of physiologically-induced fMRI signal components is a common approach in physiological noise cleaning methods (CORSICA (Perlbarg et al., 2007), CompCor (Behzadi et al., 2007), HighCor (Curtis and Menon, 2014) and PHYCAA (Churchill et al., 2012)). Their obvious advantage is the avoidance of external recordings as required by informed methods, such as RETROICOR. The dual regression scheme also allows the reconstruction of the power spectra in a data-driven manner. A first estimate of the explanatory variables can be made from the spectrally separable regimes of cardio-respiratory fluctuations. To do this we assign the voxel's EPI spectrum between 0.2 and 0.6 Hz as our initial guess for the respiratory spectrum and above 0.6 Hz for the cardiac spectrum. The subsequent dual regression iteration allows any frequency range to be taken up by the refined spectra. Any higher-order harmonics of respiration-induced signals above 0.6 Hz, as well as aliases and lower frequency cardiac-induced signals between $f_{\text{min}}$ and 0.6 Hz, can reappear in the iterated spectra. The ability of the dual regression approach, in particular the data-driven variant, to detect real signals was tested through simulations described in the Supplementary Material S1. A MATLAB example script with our dual regression function is available for download here.^3^

## Data and post-processing

### MR data

21 subjects, between the ages of 19 and 77, were scanned on a 7T whole-body scanner (MAGNETOM 7T, Siemens, Erlangen, Germany) using a 1 channel Tx/32 channel Rx head coil (Nova Medical, Wilmington, MA, USA) under institutional ethics approval. Subjects were recruited from an existing volunteer pool. When included into this pool, each volunteer received an initial brain MRI at 3T comprising at least a 3D T1-weighted scan and a FLAIR scan, which were screened for accidental findings by an experienced physician. Additional exclusion criteria were any form of known neurological condition and hypertension. Subjects had their blood pressure measured on the day of the scan to confirm their systolic pressure to be below 140 mmHg, which we used as the upper limit for normal blood pressure. Physiological traces were recorded at 1 kHz throughout the scans using a respiratory pad and a pulse-ox that was attached to the index finger (BioPac Systems, Goleta, CA, USA). The traces were later sub-sampled to the TR of the rs-fMRI scan.

#### Anatomical scanning for tissue segmentation

An MP2RAGE (Marques et al., 2010) dataset was acquired to create a T1-weighted (T1w) image. TR =8250 ms, echo time; TE =2.46 ms, inversion times; TI_1_/${\text{TI}}_{2}=1000/3300$ ms (non-selective inversion recovery), flip angles; FA_1_/${\text{FA}}_{2}=7^{\circ }/5^{\circ }$, nominal resolution = 0.8 mm isotropic, field of view; ${\text{FOV}}_{\text{read}}=210$ mm, ${\text{FOV}}_{\text{phase}}=100\text{\%}$, phase partial Fourier =6/8, 208 slices, acceleration factor; GRAPPA =2 (24 reference lines), bandwidth; BW =240Hz/px, scan time ≈14.5mins.

#### rs-fMR

A multiband EPI scan (Moeller et al., 2010, Feinberg et al., 2010) was acquired with TR =328 ms, TE =26 ms, FA $=33^{\circ }$, nominal resolution = 3.0 mm isotropic, ${\text{FOV}}_{\text{read}}=200$ mm, ${\text{FOV}}_{\text{phase}}=100\text{\%}$, phase partial Fourier =7/8, 28 slices, multiband factor =3, GRAPPA =3 (24 reference lines), BW =1994Hz/px, inter-echo spacing; ESP =0.68ms, 2200 vol, scan time ≈12mins. The ratio of physiological to thermal noise increases with field strength and increases approximately linearly with voxel volume in grey matter (Triantafyllou et al., 2005). Therefore we chose a rather “low” voxel resolution for 7T of 3 mm isotropic. However, the higher voxel volume leads to greater partial volume effects. A subsequent age bias, expected from normal aging atrophy, needs to be accounted for in the statistical analysis (see Analysis section). A separate field map was acquired for distortion correction.

### Post-processing

#### Tissue segmentation

The T1w scan was run through FSL's *fsl_anat* pipeline. It performs skull stripping, bias field correction and calculates partial volume estimate (PVE) maps for tissue segmentation (Smith et al., 2004). The EPI scan was registered to the T1w image and the inverse transformation was then applied to the PVE maps for analysis in functional space. Grey and white matter masks were created from binarising at a minimum PVE value of 0.7 to reduce partial volume effects.

#### rs-fMRI

The data were processed through FSL FEAT pipelines (Jenkinson et al., 2012), including skull stripping, motion correction, field map correction, registration to the T1w image and MNI standard space and de-meaning. The data were not spatially smoothed.

### Analysis

#### Dual regression

The post-processed data were read into MATLAB, R2016a (Mathworks, Nattick, USA) to perform the subsequent analysis.^4^ Dual regressions were run separately in the grey and white matter masks. The dual regression iteration was stopped once the sum of absolute differences between two iterated refined spectra was below 0.01 $\left(\sum_{f_{i}=0.2}^{f_{\text{max}}}|X^{n}\left(f_{i}\right)-X^{n+1}\left(f_{i}\right)|<0.01\right)$. The difference in the spectral amplitudes at the dominant^5^ cardiac frequency was calculated for the different approaches: to compare the external and the informed dual-regressed spectra i.e. $X_{c,info,WM}\left(f_{c}\right)-X_{c,ext}\left(f_{c}\right)$; to compare the informed and data-driven dual regressed spectra i.e. $X_{c,info,WM}\left(f_{c}\right)-X_{c,dd,WM}\left(f_{c}\right)$; and to compare between grey and white matter $X_{c,info,GM}\left(f_{c}\right)-X_{c,info,WM}\left(f_{c}\right)$. The analogue calculation was done for the dominant respiratory component $f_{r}$. A student's paired *t*-test was performed for all comparisons. Further we calculated the overlap of the informed and data-driven PE maps.

#### Age effects of physiological contributions to EPI power

We calculated the average PE-values in each subject and tissue. To ensure that we only calculate the average from voxels that indeed show significant modulation by the cardiac cycle and respiration we thresholded PE maps at a maximum p-value of 0.01. To account for age-related differences in partial volume effects we included a voxel-wise PVE weighting in the calculation of the PE averages. Putting this together, the tissue-specific average PE was calculated as

(5) $$\langle \beta \rangle =\sum_{\begin{array}{c} \text{PVE}>0.7\\ p_{\beta }<0.01 \end{array}}\beta \cdot \text{PVE}/\sum_{\begin{array}{c} \text{PVE}>0.7\\ p_{\beta }<0.01 \end{array}}\text{PVE},$$

With β$=\alpha ,\beta_{c},\beta_{r}\;.$

We further calculated the spatial extent, defined as the ratio of significantly modulated tissue mask volume to total tissue mask volume

(6) $$R=\sum_{\begin{array}{c} \text{PVE}>0.7\\ p_{\beta }<0.01 \end{array}}\text{PVE}/\sum_{\text{PVE}>0.7}\text{PVE}$$

(7) $$\text{With}\;\text{R}\;=R_{r},R_{c}.$$

We ran partial correlations between age and $\langle \beta_{c}\rangle ,\langle \beta_{r}\rangle ,R_{c},\;and\;R_{r}$ within both grey and white matter masks using different sets of covariates. To remove the coupling effect between $\beta_{c}$ and $\beta_{r}$ (as mentioned in the Methods section) we included the complementary parameter as a covariate of no interest. We further used the dominant cardiac frequency (i.e. cardiac rate) as a covariate for the mean cardiac PE, $\langle \beta_{c}\rangle$, and for the spatial extent, $R_{c}$. Similarly, we used the dominant respiratory frequency as a covariate for the mean respiratory PE, $\langle \beta_{r}\rangle$, and for the spatial extent, $R_{r}$. Finally, we included the absolute displacement (calculated by MCFLIRT as the mean of the root-mean-squared difference between the transformation matrix of each volume to the reference volume) as a covariate for all statistical tests. All of the above analyses were performed for both approaches - the informed and the data-driven dual regression. We further split the subjects into a “younger” (19−29 yrs) and “older” (46−77 yrs) group to calculate group averaged cardiac maps in MNI standard space. Each subject's cardiac map was transformed to MNI space using FSL's *applywarp* and maps were subsequently averaged.

### Comparison with cardiac pulsatility metric

For a comparison with the previous literature we calculated the cardiac pulsatility metric as proposed by Makedonov et al. (2013). This metric is defined as the average power in a 0.04 Hz window, centred at the maximum power component that is taken from the externally measured cardiac spectrum. We calculated the cardiac pulsatility metric in all tissue masks twice, once including all voxels that counted towards the mask (as done in the original paper by Makedonov et al.) and once only including the voxels that showed a significant cardiac modulation in the dual regression (p<0.01). Partial correlation with age, while controlling for the absolute displacement from the motion correction step and cardiac rate were performed subsequently.

## Results

One subject (65 years old) exhibited multiple absolute head displacements of about the voxel dimension (3 mm) during the scan and was therefore excluded from the results.

### Dual regression

**Fig. 4:**
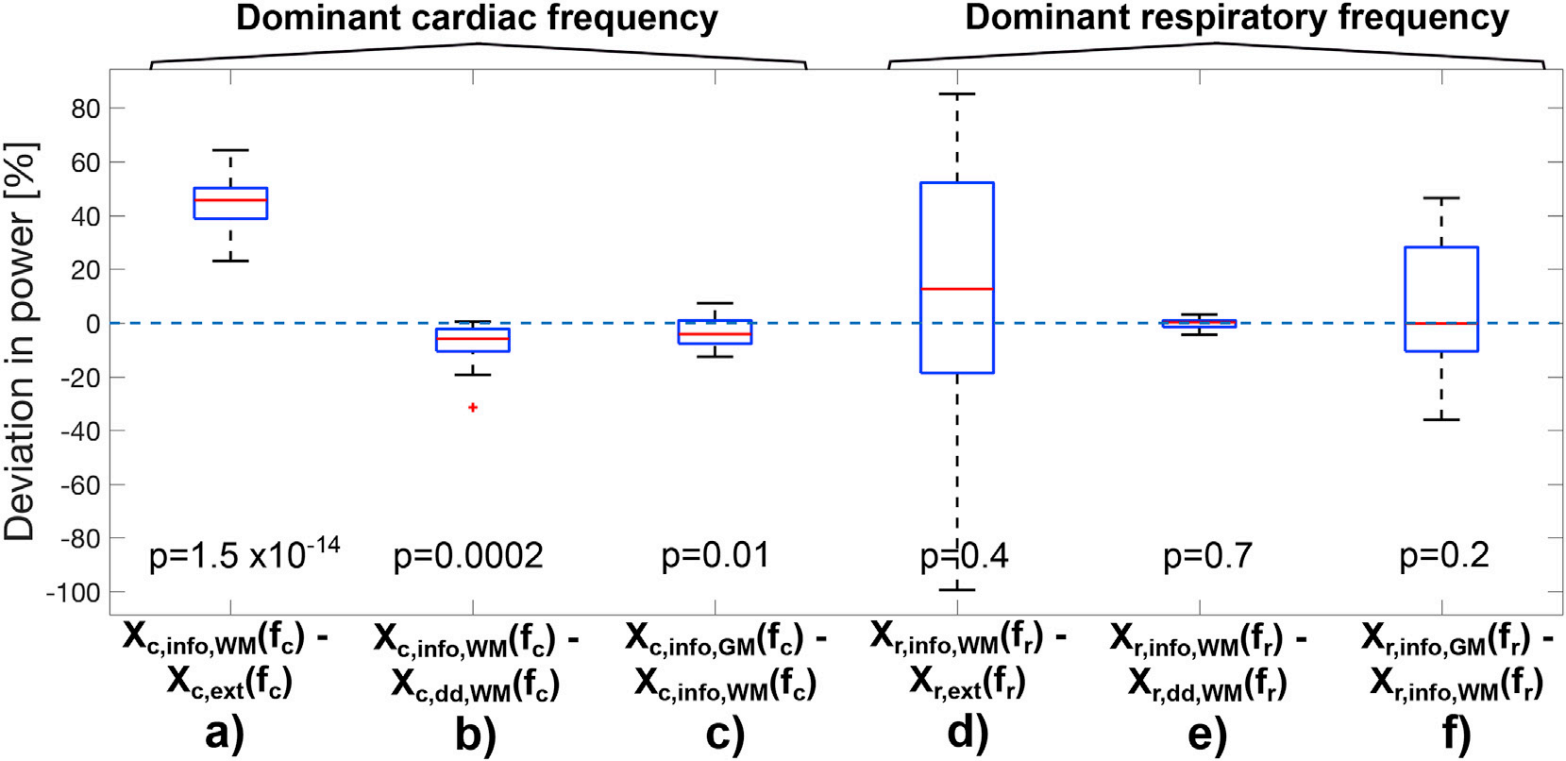
Box plots of the percentage power deviation at the dominant frequencies. These are the differences between a) the informed dual-regressed white matter cardiac spectrum and the external pulse-ox spectrum; b) the informed and data-driven dual regressed cardiac spectra in white matter; c) the informed dual-regressed cardiac spectra in grey and white matter; d) the informed dual-regressed white matter respiratory spectrum and the external respiratory spectrum; e) the informed and data-driven dual regressed respiratory spectra in white matter; f) the informed dual-regressed respiratory spectra in grey and white matter. Box plot properties: the red line is the median, the blue box marks the 25%−75% quantiles, the whiskers span the ±2.7σ range and red crosses mark outliers. P-values refer to Student's paired *t*-test.

Fig. 4 shows the percentage differences at the dominant cardio-respiratory frequencies for the different spectra. The external cardiac spectrum has systematically lower power at the dominant cardiac frequency compared to the informed dual regressed spectrum in white matter ($p=1.5\cdot 10^{-14}$, see Fig. 4a). The data-driven dual-regressed cardiac frequency power is larger compared to the informed dual regressed one (p=0.0002, see Fig. 4b). The grey matter spectrum shows a higher power amplitude than the white matter spectrum (p=0.01, see Fig. 4c). The deviation in the respiratory spectra shows a strong inter-subject as well as within-tissue variability (see Fig. 4d) and f)) and showed no systematic difference. The spatial overlap between the cardiac PE masks from the informed and data-driven dual regression was high with 99.6±0.3% in white matter and 99.7±0.4% in grey matter, respectively. The respiratory PE masks overlapped by 98.7±3.2% in white matter and by 99.8±0.3% in grey matter.

### Age effects of cardiac frequency contributions to EPI power

**Fig. 5:**
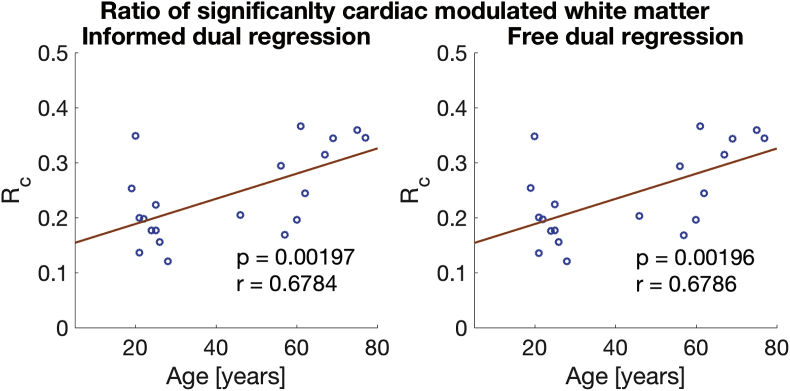
$R_{c}$, the ratio of significantly cardiac modulated white matter volume, against age. Results from the informed dual regression are in a) and from the data-driven approach in b). The scatter plots are almost identical and both regressions yield comparable results The p-values refer to a partial correlation with age, controlled for the cardiac rate and the displacement. The r-value is Pearson's correlation coefficient.

We found a significant increase of the spatial extent of the cardiac cycle-induced fluctuations, $R_{c}$, with age in white matter with p-values of 0.002 for both the informed and data-driven dual regression. The scatter plots for white matter are provided in Fig. 5 (as suggested by the similar p-values the informed and dual-regressed results are almost identical). The same statistical test did not reach a significant level in grey matter (p-values of 0.096 and 0.092).

**Fig. 6:**
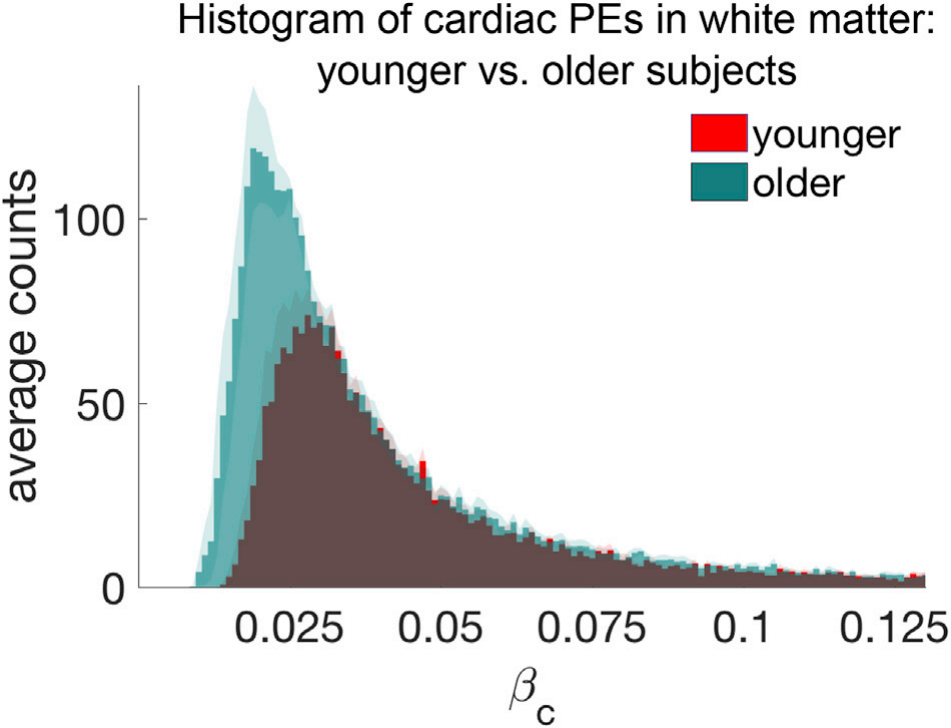
Average distribution of cardiac PE $\langle \beta_{c}\rangle$ in the younger (19−29 ys, red plot) and older (46−77 ys, green plot) subjects in white matter. The younger subjects' distribution is almost a complete subset of the older subject's distribution (the overlap is coloured in brown). The standard error of the mean is plotted in shaded colour on top of the distributions.

**Fig. 7:**
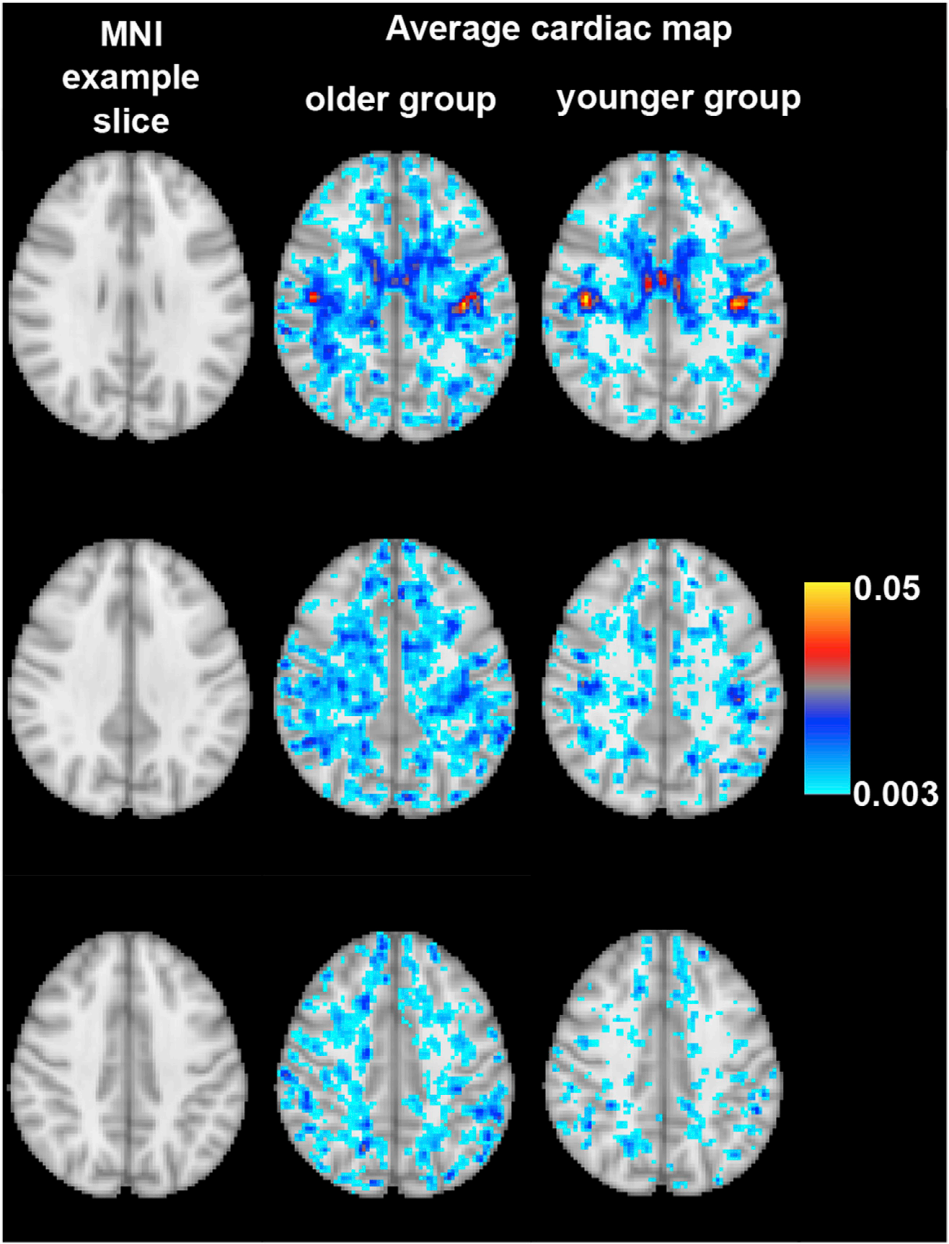
White matter cardiac *β_c_* maps in MNI space. The left column shows the MNI space slices, the middle column are the group-averaged cardiac maps for the older group and the right column are the younger group. Similar to the distribution of cardiac PEs in Fig. 6, this MNI overlay visualises how the older group has an increased spatial extent of cardiac pulsatility in the white matter.

Initial analysis suggested a decrease of the mean cardiac PE $\langle \beta_{c}\rangle$ with age in white matter. This correlation (controlled for $\langle \beta_{r}\rangle$, cardiac rate and absolute motion displacement) has a p-value of 0.026 (informed dual regression). There was no effect in grey matter (p-values of 0.27 and 0.71). Nevertheless, we suspect that the decrease in white matter is explained by the age-related increase in the volume of white matter, $R_{c}$, showing significant cardiac modulation. Indeed, a repetition of the partial correlation between $\langle \beta_{c}\rangle$ and age, whilst further including $R_{c}$ as a covariate, led to p-values of 0.59 (informed dual regression). To visualise the effect of how the increased cardiac modulation in white matter affects the average PE, we plotted the group-averaged distributions of cardiac regressors $\beta_{c}$ of the younger and older groups in Fig. 6. The plot reveals how the younger $\beta_{c}$ distribution (red) forms a subset of the older distribution (green) and $\beta_{c}$ counts increase towards smaller values for the older group. Fig. 7 shows three example slices of the group-averaged white matter cardiac maps in MNI space. In line with the distribution plots in Fig. 6 the older group shows a wider extent of cardiac modulated voxels in the white matter tissue.

We did not find any age effect for the respiratory component. Any correlation between the $\langle \beta_{r}\rangle$ or $R_{r}$ and age vanished when the covariates of no interest ($\langle \beta_{c}\rangle$, respiratory rate, absolute displacement) were controlled for. Similarly, there was no age effect for the thermal noise baseline, as would be expected.

### Comparison with cardiac pulsatility metric

The results of the cardiac pulsatility metric, when calculated from voxels in a tissue mask, showed an increase in cardiac pulsatility in white matter with age (p<0.02). Results in grey matter were not significant. Within white matter the cardiac pulsatility metric lost significance with a p-value of 0.08 when the metric was calculated from the voxels that showed significant cardiac modulation only. Similarly, the metric, when calculated from all voxels, became non-significant (p = 0.37), when the ratio of significantly cardiac modulated voxels $R_{c}$ was included in the statistical test as a covariate.

## Discussion

### Dual regression analysis

The GLM approach allows the identification of voxels with significant power contributions from physiological sources and the disentanglement of the high frequency spectrum into its thermal noise, cardiac and respiratory-related contributions. The iterative dual regression further refines this analysis and can be used to create tissue-specific cardiac and respiratory power spectra. Indeed, we found that these tissue-specific cardiac spectra differ substantially from the external pulse-ox spectrum. Specifically, we found that the dominant cardiac frequency carries more power than the pulse-ox spectrum. Cardiac cycle-induced fluctuations in the EPI time series are caused by multiple mechanisms, from inflow effects, partial volume fluctuations to non-rigid brain motion (Poncelet et al., 1992, Zhong et al., 2009, Soellinger et al., 2009, Viessmann et al., 2017). As such, the non-ideal representation of the brain's BOLD response to the pulse-ox waveform at the index finger is expected. Comparing the tissue-specific cardiac spectra we found that the grey matter spectrum carries more power at the dominant frequency compared to white matter. There was a substantial mismatch between the respiratory pad and dual regression spectra. However, this mismatch was not systematic and showed a strong variability between subjects. There was also a strong variability between grey and white matter-specific spectra. In contrast to the cardiac component the respiratory component arises from more diverse sources. These include the susceptibility changes with air volume changes and respiratory rate, PET-CO_2_ fluctuations that alter blood flow velocities and modify the haemodynamic BOLD response, thoracic pressure changes that influence venous return flow velocities and further bulk motion. Another explanation for the variability observed is high-frequency resting-state network activity above 0.1 Hz (Chen and Glover, 2015, Gohel and Biswal, 2015).

### Informed vs. data-driven dual regression

The broadband spectra offered enough degrees of freedom to reach comparable results for a data-driven dual regression. The data-driven cardiac spectrum had smaller power at the dominant frequencies, whereas the respiratory spectra were negligibly different. Spatial PE maps were in good agreement. The data-driven approach thus offers an alternative for this power spectrum analysis where external recordings are not available.

### Age effects of cardiac frequency contributions to EPI power

Here, we only found a significant age effect in the white matter mask, with an increase in the spatial extent of cardiac cycle-related EPI signal fluctuations with age. The most prominent source of these signal fluctuations is the inflow effect of arterial blood, but further intra-voxel partial volume exchanges contribute to the signal pulsatility (Viessmann et al., 2017). The latter is the more likely effect in white matter, where blood flow velocities are too slow to produce an inflow effect, but intra-voxel partial volume fluctuations between tissue, blood and interstitial fluid likely alters the EPI time course over the cardiac cycle. Flow pulsatility has been reported to increase with age in the larger cerebral arteries (Tarumi et al., 2014) and it is suspected that the transformation of the pulsatile flow to a steady blood flow in the microcirculation declines with age-related arterial wall stiffening (O'Rourke and Hashimoto, 2007), which potentially causes more volume pulsatility deeper in the white matter tissue.

We further calculated the recently proposed cardiac pulsatility metric by Makedonov et al. (2013). Similar to their report we found an increase of the metric with age in white matter. However, this effect was explained by the age-related spatial increase of voxels that showed significant cardiac power modulation. As such, our findings do not support the theory of an elevated cardiac contribution to the EPI power with age in white matter, but only an enhancement in the spatial extent of the effect. Additionally, a cardiac pulsatility metric, based on a fixed spectral window width, is likely biased to heart rate variability and also to the shape of the cardiac spectrum. Heart rate variability is thought to decrease with age (O'Brien et al., 1986) and the cardiac spectral width should subsequently decrease (i.e. more power is likely transmitted via fewer frequency components in the older subjects). Our proposed dual regression analysis is independent of the spectral shape. Further, it excludes voxels from statistical analysis that do not show significant physiological EPI power.

## Conclusion

We introduced an iterative dual regression methodology that maps and quantifies physiological contributions to the EPI spectrum. Using this analysis framework we found a spatial increase of cardiac cycle-induced power in white matter with age. Changes in cardiac pulsatility in white matter with healthy aging, small vessel disease and Alzheimer's disease have been suggested in the literature. However, the number of reports is small, and here we only studied a small number of subjects. The data-driven dual regression approach might offer the ability to apply similar analysis on already existing fast rs-fMRI data sets, even when external physiological recordings are missing.

## Footnotes

1 here, we refer to “pulsatility” as the fluctuation of a variable around its mean value over the course of the cardiac period.

2 average spectrum, derived from all voxels.

3 https://github.com/OliviaViessmann/Dual_Regression.

4 A MATLAB test script with our dual regression function is available for download at https://github.com/OliviaViessmann/Dual_Regression.

5 the component with the highest power amplitude. i.e. at the cardiac rate.
