# Supplementary material for: Dual regression physiological modeling of resting-state EPI power spectra: Effects of healthy aging
Source: Neuroimage. 2019 Feb 15;187:68–76. doi: 10.1016/j.neuroimage.2018.01.011 (PMC6414402; doi:10.1016/j.neuroimage.2018.01.011)
Supplement: Supplementary [file mmc1.pdf]

Supplementary Material

S1: Code testing of dual regression via simulations

We tested the ability of the data-driven dual regression scheme to detect signals through simulations. An input test image was created from an example slice of the T1-weighted MNI standard image. The grey scale image was converted to RGB-scale, the goal being to use each channel as a weight for synthesized voxel signals with the R-channel serving as the noise contribution, the G-channel for cardiac and the B-channel for respiratory contributions, i.e. $(R, G, B) \rightarrow (\alpha, \beta_c, \beta_r)$. Grey matter voxels were assigned similar magnitude G- and B-values (≈ 0.5). White matter voxels were assigned high G-values and much smaller B-values (≈ 0.05). The CSF edge voxels were assigned high B-values and lower G-values. The resulting test image can be found in Figure SF1 b). The physiological recording of an example subject was used to create exemplar cardiac and respiratory traces $S_c(t)$ and $S_r(t)$. The amplitudes of the cardiac and respiratory traces were scaled to unit amplitude ranges. A random noise signal $S_n(t)$ was created for every voxel. Each voxel was assigned the total signal of $R \cdot S_n(t) + G \cdot S_c(t) + B \cdot S_r(t)$ (Figure SF1a)). The signal was then sub-sampled to the TR used in our experiments. This artificial data set thus represents a whole-brain consisting of varying physiological noise contributions. A data-driven dual regression was run on this synthesized data set with equivalent parameters as used in our data analysis (p-value of 0.01 and a convergence level of 0.01).The output PE maps were then compared to the input RGB test image. Figure SF1 c) shows the resulting output and d) the mean absolute error across all channels (i.e. PE values). The input and output images match by varying degrees, with a poorer match in the "greenest" and "bluest" parts of the image. These voxels are predominantly single channel weighted and higher mismatches are the result of the inter-dependence (enhancement/suppression) of PE values due to normalisation constraints as discussed in the main manuscript. For that reason it is important to add each PE value as a control variable in the statistical analysis.

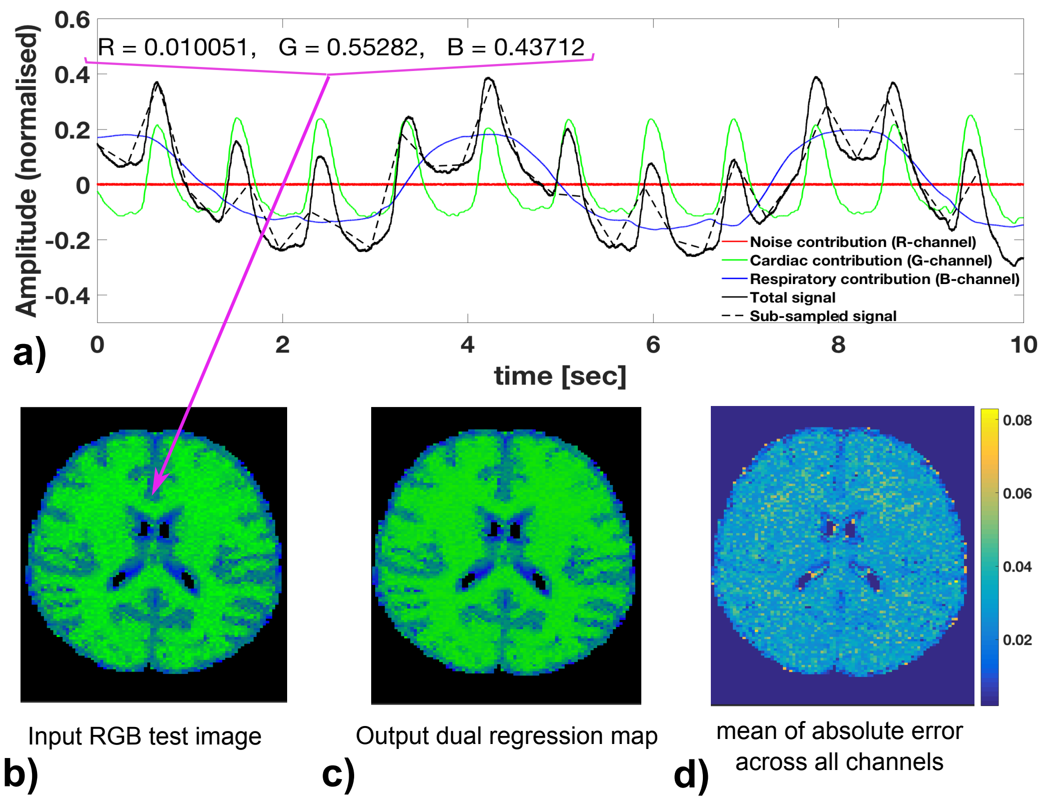

Figure SF1: Overview of the simulation for code testing. Artificial signals were created as the sum of a random noise, cardiac and respiratory signal, weighted by R-, G-, and B-values respectively (a). The RGB values were derived by colour-coding an exemplar MNI standard space image (b). The synthesised data volume was then run through the data-driven dual regression to yield the equivalent $\alpha-, \beta_c$ and β_r maps that were recombined to form an output RGB image (c) for comparison with the original (d).
